# An asymptotic theory for waves guided by diffraction gratings or along microstructured surfaces

**DOI:** 10.1098/rspa.2013.0467

**Published:** 2014-01-08

**Authors:** T. Antonakakis, R. V. Craster, S. Guenneau, E. A. Skelton

**Affiliations:** 1 Department of Mathematics, Imperial College London, London SW7 2AZ, UK; 2 European Organization for Nuclear Research, CERN 1211, Geneva 23, Switzerland; 3 Institut Fresnel, UMR CNRS 7249, Aix-Marseille Université, Ecole Centrale Marseille, 13013 Marseille, France

**Keywords:** plasmonics, homogenization, Rayleigh–Bloch waves

## Abstract

An effective surface equation, that encapsulates the detail of a microstructure, is developed to model microstructured surfaces. The equations deduced accurately reproduce a key feature of surface wave phenomena, created by periodic geometry, that are commonly called Rayleigh–Bloch waves, but which also go under other names, for example, spoof surface plasmon polaritons in photonics. Several illustrative examples are considered and it is shown that the theory extends to similar waves that propagate along gratings. Line source excitation is considered, and an implicit long-scale wavelength is identified and compared with full numerical simulations. We also investigate non-periodic situations where a long-scale geometrical variation in the structure is introduced and show that localized defect states emerge which the asymptotic theory explains.

## 1. Introduction

It has been known for many years that surface waves, that is, waves propagating along a surface, and exponentially decaying in amplitude perpendicular to the surface, are created by geometrical periodic corrugations, or perturbations, to the surface [1–3] in situations where a surface wave would otherwise not exist. Such surface waves also occur for diffraction gratings and for trapped modes in waveguides; these are all very similar problems mathematically [4,5] and differ just in their setting. These surface waves have been discovered in many different areas of wave mechanics and go under names such as edge waves [6] for water waves localized to periodic coastlines, spoof surface plasmon polaritons (SPPs) [7,8] in modern applications of plasmonics, array-guided surface waves [9] in Yagi–Uda antenna theory, Rayleigh–Bloch surface waves [5,10] for diffraction gratings among other areas: we will call them Rayleigh–Bloch waves as surface waves are typically called Rayleigh waves, and Bloch waves arise owing to periodicity. They can also be identified in lattice defect arrays, in discrete settings [11], and are ubiquitous across wave mechanics, it is important to clearly delineate them from surface waves, such as Rayleigh waves, that are present in the absence of periodic geometrical features and which arise owing to material mismatch or from wave mode coupling at the surface.

Naturally, as these are eigenfunctions of a diffraction grating, they have implications for the uniqueness of solutions and they have been the subject of numerous existence studies [10,12,13] with the conclusion that they are a generic property of periodic surfaces and gratings that have Neumann boundary conditions; the non-existence for Dirichlet cases for the wave equation is shown in Wilcox [10].

As well as being ubiquitous in wave mechanics, Rayleigh–Bloch waves are important in applications; their dispersion characteristics can be carefully tuned by altering only the geometry as in SPPs [8], or are important through the coupling of incident waves into Rayleigh–Bloch waves causing near resonant effects for finite arrays as in water waves [14,15]. These effects, and in particular the possibility to tune or detune them, rely upon being able to simulate and determine dispersion characteristics; there is advantage in being able to represent and model them using an effective medium approach that replaces the microstructure.

The classical route to replace a microstructured medium with an effective continuum representation is homogenization theory, and for bulk media this is detailed in many monographs, for instance Sanchez-Palencia [16], Bakhvalov & Panasenko [17], Bensoussan *et al.* [18], Panasenko [19], and essentially relies upon the wavelength being much larger than the microstructure which is usually assumed to be perfectly periodic: the theory has been very versatile and has been widely applied. Naturally, there were extensions of this theory to surfaces, notably by Nevard & Keller [20], again with the wavelength limitation, unfortunately this long-wave low-frequency limit is not particularly useful at the high frequencies used in applications such as photonics [21] and plasmonics [22,23]; this motivated the development of high-frequency homogenization (HFH) in Craster *et al.* [24]. HFH breaks free of the low-frequency long-wave limitation and, for bulk media, creates effective long-scale equations that encapsulate the microstructural behaviour, which can be upon the same scale as the wavelength, through integrated quantities that are no longer simple averages. The methodology relies upon there being some basic underlying periodic structure, so that Bloch waves and standing wave frequencies encapsulate the multiple scattering between elements of the microstructure on the short scale, and this is then modulated by a long-scale function that satisfies an anisotropic frequency-dependent partial differential equation; the technique has been successfully applied to acoustics/electromagnetics [25,26], elastic plates that support bending waves [27], frames [28] and to discrete media [29]. The advantage of having an effective equation for a microstructured bulk medium or surface is that one need no longer model the detail of each individual scatterer, as they are subsumed into a parameter on the long scale, and attention can then be given to the overall physics of the structure and one can identify, or design for, novel physics.

The HFH theory of Craster *et al.* [24] is not alone: there is considerable interest in creating effective continuum models of microstructured media, in various related fields, that break free from the conventional low-frequency homogenization limitations. This desire has created a suite of extended homogenization theories originating in applied analysis, for periodic media, called Bloch homogenization [30–33]. There is also a flourishing literature on developing homogenized elastic media, with frequency-dependent effective parameters, also based upon periodic media as in Nemat-Nasser *et al.* [34]. Those approaches notwithstanding, our aim here is to extend the HFH theory to microstructured surfaces and obtain frequency-dependent effective surface conditions that capture the main features of the surface waves that exist.

Our aim herein is to generate a surface HFH theory for structured surfaces in the context of periodic surfaces. Importantly, one can modify the theory, as performed for bulk waves in Craster *et al.* [25], Antonakakis & Craster [27] and Makwana & Craster [35], to pull out defect states associated with non-periodic variation. It is also important to note that the HFH theory has a deep connection with the high-frequency long wavelength near-cut-off theory of waveguides [36], and the defect states are related to localization by deformed waveguides [37–39]. We also naturally extend the HFH theory to diffraction gratings. In §2, the theory is created culminating in the effective equation that encapsulates the surface behaviour. Illustrative examples, in §3, then show the efficacy of the methodology versus the dispersion relations found numerically. An interesting practical situation is where some geometrical variation occurs, then one expects the possibility of trapped modes along the structure occurring at a set of discrete frequencies, and we consider a comb-like structure where the teeth have varying length in §4; the asymptotic theory is compared with full numerical simulations. Finally, concluding comments and remarks are drawn together in §5.

## 2. General theory

**Figure 1.**
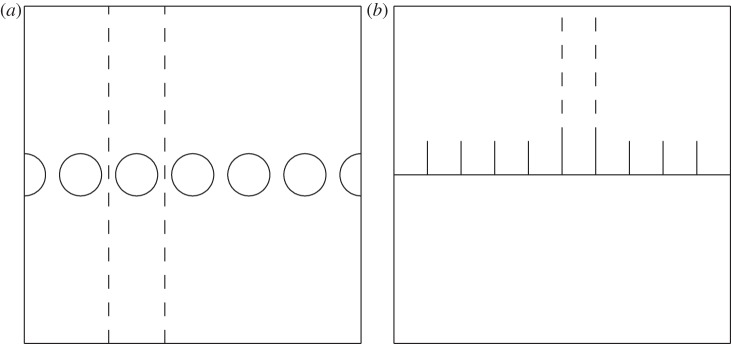
A diffraction grating of cylinders shown in (*a*) and (*b*) shows a periodic ‘comb’ surface that supports SSPs. Both (*a*,*b*) have the elementary strip shown as the dashed lines.

For perfect infinite linear arrays, diffraction gratings or surface structures arranged periodically, one focuses attention on a single elementary strip of material that then repeats (see figure 1 for illustrative cases); quasi-periodic Floquet–Bloch boundary conditions describe the phase-shift across the strip as a wave moves from strip to strip through the material. Rayleigh–Bloch waves are special as they consist of waves that also decay exponentially in the perpendicular direction away from the array. Dispersion relations then relate the Floquet–Bloch wavenumber, the phase-shift, to frequency. Although the problem is truly two-dimensional, the assumption of exponential decay in the perpendicular direction renders it quasi-one dimensional with the wavenumber remaining scalar; this contrasts with the theory of Bloch waves in photonic crystals [21] where a vector wavenumber and the Brillouin zone are more natural descriptions.

We generate an asymptotic theory and importantly we take Neumann boundary conditions on the lattice, or surface, where physically this can be considered as transverse electric (TE) polarization for a perfectly conducting surface which is a good model for microwaves [40].

A time harmonic dependence of propagation 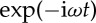, with frequency *ω*, is assumed throughout, and henceforth suppressed, and after non-dimensionalization of the frequency one arrives at
2.1
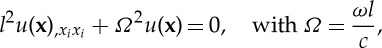
where *l* is the length scale of the microscale and *c* is the wavespeed, as the governing equation of interest. We consider the half-space 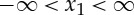, 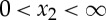, and for the grating extend to 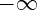 in *x*_2_, where *x*_1_ and *x*_2_ are in the horizontal and vertical directions, respectively. In (2.1), *Ω* is the non-dimensional frequency, and *u* is the out-of-plane displacement in elasticity or the *H*_3_ component of the magnetic field in TE polarization.

The two-scale nature of the problem is incorporated using small and large length scales to define two new independent coordinates, namely *X*=*x*_1_/*L*, and (*ξ*_1_,*ξ*_2_)=(*x*_1_,*x*_2_)/*l*. The implicit assumption is that there is a small scale, characterized by *l* defined earlier, and a long scale characterized by *L* that represents a characteristic length scale of the whole grating where *ϵ*=*l*/*L*≪1. As the structure is quasi-one-dimensional, with the mismatch in the scales being just along the structure, we introduce only a single long-scaled variable in *X*; we do not introduce a long-scale *Y* in the *x*_2_-direction as it is redundant.

Under this rescaling, equation (2.1) then becomes,
2.2
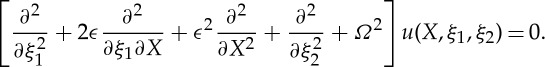
Standing waves, that exponentially decay perpendicular to the surface/grating, can occur when there are periodic (or anti-periodic) boundary conditions across the elementary strip (in the ***ξ*** coordinates) and these standing waves encode the local information about the multiple scattering that occurs by the neighbouring strips. The asymptotic technique we create is a perturbation about these standing wave solutions, as these are associated with periodic and anti-periodic boundary conditions, which are, respectively, in-phase and out-of-phase waves across the strip, the conditions on the short-scale ***ξ*** on the edges of the strip, ∂*S*_1_, are known:
2.3
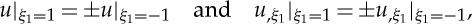
where *u*_,*ξ*_*i*__ denotes differentiation of *u* with respect to variable *ξ*_*i*_ and with the +,− for periodic or anti-periodic cases, respectively. There is therefore a local solution on the small scale that incorporates the multiple scattering of a periodic medium and that will then be modulated by a long-scale function that satisfies a differential equation. Typically, the periodic case corresponds to long-waves relative to the structure—this case is not particularly interesting and is captured by conventional low-frequency homogenization. We therefore concentrate upon the anti-periodic case.

We pose an ansatz for the field and the frequency,
2.4
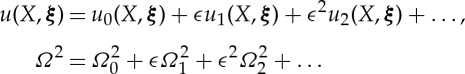
The *u*_*i*_(*X*,***ξ***)'s adopt the boundary conditions (2.3) on the short-scale, with the minus sign for anti-periodicity, on the edge of the strip. An ordered hierarchy of equations emerges in powers of *ϵ*, and is treated in turn
2.5
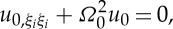

2.6
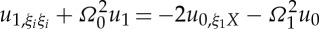

and
2.7
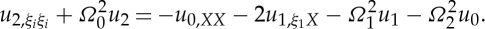
The leading-order equation (2.5) is independent of the long-scale *X* and is a standing wave on the elementary strip existing at a specific eigenfrequency *Ω*_0_ and has associated eigenmode *U*_0_(***ξ***;*Ω*_0_), modulated by a long-scale function *f*_0_(*X*), and so we expect to get an ordinary differential equation (ODE) for *f*_0_ as an effective boundary, or interface, condition characterizing the grating when viewed from afar. To leading order
2.8
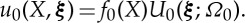
The entire aim is to arrive at an ODE for *f*_0_ posed entirely upon the long-scale, but with the microscale incorporated through coefficients that are integrated, not necessarily averaged, quantities. *f*_0_ represents the amplitude modulation of short-scale oscillations over the grating over wavelengths commensurate with the size of *L*.

Before we continue to next order, equation (2.6), we define the Neumann boundary conditions on the inclusions ∂*S*_2_, or the microstructured surface, as
2.9
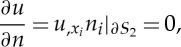
using Einstein's notation for summation over repeated indices, and where **n** is the outward pointing normal, which in terms of the two scales and *u*_*i*_(*X*,***ξ***) become
2.10
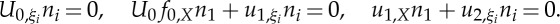
The leading-order eigenfunction *U*_0_(***ξ***;*Ω*_0_) must satisfy the first of these conditions and it is relatively straightforward to extract this either numerically, as we do later, or using semi-analytic methods such as the residue calculus technique [2].

Moving to the first-order equation (2.6), we invoke a solvability condition by integrating over the elementary strip *S*, which is on the short-scale ***ξ***, the product of equation (2.6) and *U*_0_ minus the product of equation (2.5) and *u*_1_/*f*_0_(*X*). The result is that the eigenvalue *Ω*_1_ is identically zero.

We then solve for *u*_1_=*f*_0,*X*_*U*_1_(***ξ***), so *U*_1_ satisfies
2.11
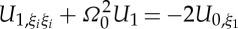
subjected to the boundary condition
2.12
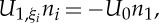
on ∂*S*_2_. Again, solutions can be found numerically or using semi-analytic methods such as multi-poles and lattice sums [41], or other numerical methods that have proved their usefulness in diffraction theory [42].

Going to the second-order, a similar solvability condition to that used at the first-order is applied using equation (2.7); after some algebra, we obtain the desired ODE for *f*_0_
2.13
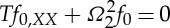
posed entirely on the long-scale *X*. The coefficient *T* is constructed from integrals over the elementary strip in ***ξ*** and is ultimately independent of ***ξ***. The formula for *T* is
2.14
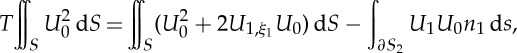
which using Green's theorem, with vector field **F**=(*U*_1_*U*_0_,0), simplifies to,
2.15
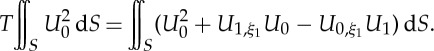
For an infinite grating of cell width 2 (*l*=1), the Bloch grating *f*_0_ needs to have symmetric boundary conditions for *κ*=*π*/2 and therefore, 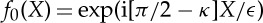 and equation (2.13) simplifies to 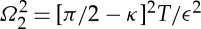 and from (2.4) the asymptotic dispersion relation relating frequency, *Ω*, to Bloch wavenumber, *κ*, is
2.16
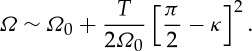
In (2.16), *T* is invariably negative, cf. table 1 for some illustrative values, as is 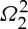, the latter should not be confused with having negative frequencies as it is merely a frequency perturbation. Therefore, if the surface or grating supports Rayleigh–Bloch waves, then they are represented as an effective string or membrane equation (2.13) where the effective stiffness (or effective inverse of permittivity in the context of photonics) of the string is *T*; all the microstructural and geometrical information is encapsulated in this asymptotic result and one can then extend it to be used for finite arrays or for slightly non-periodic arrays, or forced problems, etc., but our aim here is to now demonstrate that this theory is well founded.

**Table 1.** The four standing wave frequencies for the comb-like structure with *a*=7 (cf. figure 2*d*), together with associated values for *T*.

*T*	*Ω*_0_
−0.006485497624108	0.210161050669707
−0.067470169867289	0.629209426388598
−0.280897588912595	1.043323585456635
−2.350025233704123	1.440535862845912

### (a) The classical long-wave zero-frequency limit

The current theory simplifies dramatically in the classical long-wave, low-frequency, limit where *Ω*^2^∼*O*(*ϵ*^2^), this is a periodic case on the short-scale: *U*_0_ becomes uniform, and without loss of generality, is set to be unity over the elementary strip. The final equation is again (2.13) but in limiting form and using a rectangular strip of height y* for S*, *T* simplifies to
2.17
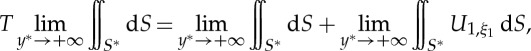
where S*=*S*∩*C*|{*C*=[−*l*,*l*]×[−y*,y*]}. *U*_1_*i*__ satisfies the Laplacian *U*_1,*ξ*_*i*_*ξ*_*i*__=0 and *U*_1_ has boundary conditions *U*_1,*ξ*_*i*__*n*_*i*_=−*n*_1_ on ∂*S*_2_. Rearranging equation (2.17) yields
2.18
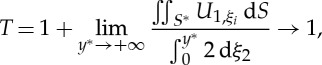
from which *Ω*=*κ*, and thus the light-line of unit slope emerging from the origin arises asymptotically.

### (b) A dynamic characteristic length scale

As we see later, in §3, equation (2.16) is an excellent asymptotic approximation for the dispersion diagrams of such gratings that verifies the validity of HFH. Ultimately, one wishes to homogenize a periodic, or nearly periodic, surface and this is achieved with equation (2.13) transformed back in the original coordinates together with the replacement of *Ω*_2_ using the asymptotic expansion in equation (2.4). The effective medium equation resulting from such operations is
2.19
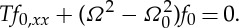
The solutions of equation (2.19) are harmonic with argument 
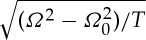
 provided *Ω*<*Ω*_0_ and *T*<0. It is now clear that if the excitation frequency is slightly away from the standing wave frequency, then an oscillation will emerge with wavelength 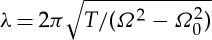 which will represent a characteristic length scale for such an infinite periodic medium. That length scale not only depends on the excitation and standing wave frequencies but also on the homogenized parameter *T* that represents dynamically averaged material parameters (e.g. the inverse of effective permittivity in photonics). Therefore, one observes highly oscillatory behaviour with each neighbouring strip out-of-phase but modulated by a long-scale oscillation of wavelength *λ*/2 reminiscent of a beat frequency but induced by the microstructure; the *λ*/2 arises as one observes both *f*_0_ and −*f*_0_.

## 3. Illustrative examples

We now illustrate the theory using linear arrays of cylinders, split ring resonators (SRRs) and a comb-like surface structure as these are exemplars of the situations seen in practice. In order to model infinite media in finite-element software, we use perfectly matched layers adapted for acoustics [43].

### (a) The classical comb

An early example for which Rayleigh–Bloch waves were found explicitly is that of a Neumann comb-like surface consisting of periodic thin plates of finite length, *a*, perpendicular to a flat wall and distant by 2*l* from each other. This was initially studied by Hurd [2] with later modifications by DeSanto [44], Evans & Linton [6] and Evans & Porter [45]. It is a canonical example and can be considered as a diffraction grating if extended to the negative half-plane by reflection symmetry.

**Figure 2.**
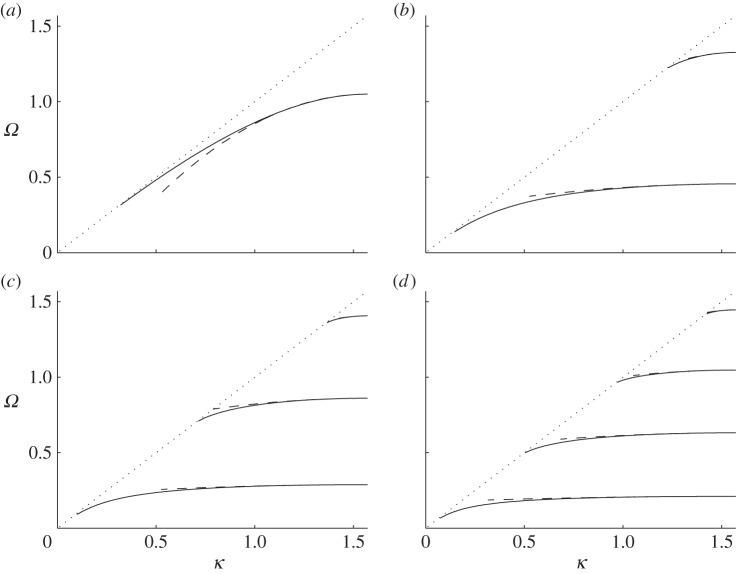
The dispersion branches for the comb-like structure. Solid lines are from (3.2), and the light-line *Ω*=*κ* is the dotted line. (*a*–*d*) Are for *a*=1, 3, 5 and 7, respectively. Asymptotics from HFH (2.16), with *T* given in table 1 for *a*=7 (*d*), are depicted as dashed curves.

**Figure 3.**
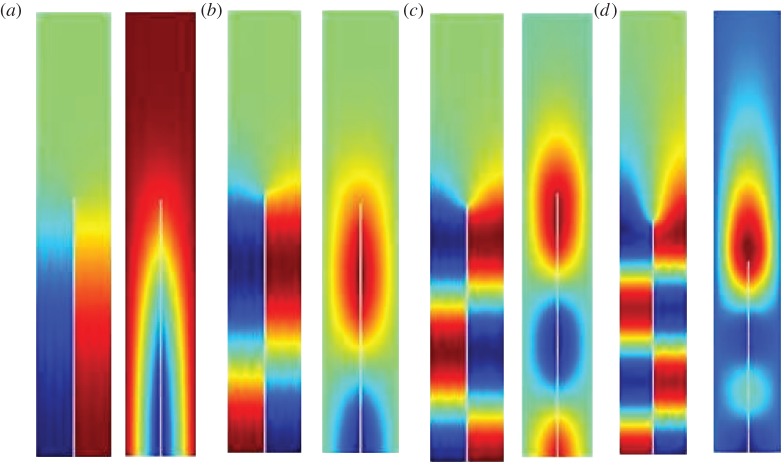
The eigenfunctions *U*_0_ and *U*_1_ shown for the comb-like structure with *a*=7 (cf. figure 2*d*). These are for the standing wave frequencies *Ω*_0_ in table 1 with (*a*–*d*) for ascending *Ω*_0_. In each panel, *U*_0_ is shown on the left and *U*_1_ on the right. (Online version in colour)

We will concentrate upon non-embedded Rayleigh–Bloch waves in *Ω*<*κ* and Hurd's dispersion relation
3.1
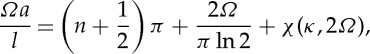
where
3.2
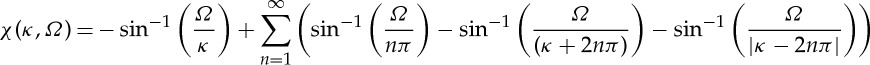
provides a highly accurate approximation; dispersion branches are shown in figure 2 using Hurd's formulae. There exists an even more accurate result from Evans & Linton [6] which is virtually indistinguishable from that of Hurd, and it is possible, as we also do here, to use finite elements to model the comb numerically, the only detail of note is that the comb teeth have finite width of 0.05 in the finite-element simulations to avoid any numerical issues at the tip of the teeth, and all these methods give coincident results. The width of the cell is taken to be 2, so that the small scale *l* is set to *l*=1. This convention will be used in all subsequent illustrations. The dispersion equations (3.2) come from a Fourier series approach and we also investigate this approach numerically and provide results in tables 2 and 3. The parameter *a* is the length of the tooth and the curves are locally quadratic near *π*/2 as we expect from (2.16); clearly, the HFH asymptotics provide an excellent representation of the dispersion curves close to the standing wave frequency as illustrated by the dashed curves in figure 2. The standing wave frequencies *Ω*_0_ and the effective parameter *T* are given in table 1 for the case *a*=7. Increasing *a* corresponds to more dispersion curves appearing and the eigensolutions for *a*=7 are shown in figure 3 together with their *U*_1_ counterparts, and the reason for the increasing number of surface modes is immediately apparent being intimately connected with the number of modes the open waveguide supports. The *U*_0_ modes decay rapidly as they exit the open waveguide particularly for the lowest standing wave frequencies.

**Table 2.** The four standing wave frequencies for the comb-like structure with *a*=7 calculated from Fourier series expansion with the convergence established by increasing the number of modes used.

*N*	2	5	10	20	40
*Ω*_0_	0.2085	0.2101	0.2106	0.2108	0.2109
*Ω*_0_	0.6244	0.6290	0.6305	0.6312	0.6315
*Ω*_0_	1.0355	1.0431	1.0454	1.0466	1.0471
*Ω*_0_	1.4307	1.4404	1.4433	1.4447	1.4454

**Table 3.** The values of *T* associated with the four standing wave frequencies for the comb-like structure with *a*=7 calculated from Fourier series expansion with the convergence established by increasing the number of modes used.

*N*	2	5	10	20	40
*T*	−0.0068	−0.0066	−0.0066	−0.0066	−0.0066
*T*	−0.0704	−0.0687	−0.0686	−0.0686	−0.0686
*T*	−0.2877	−0.2843	−0.2849	−0.2856	−0.2860
*T*	−2.2022	−2.3602	−2.4267	−2.4628	−2.4816

**Figure 4.**
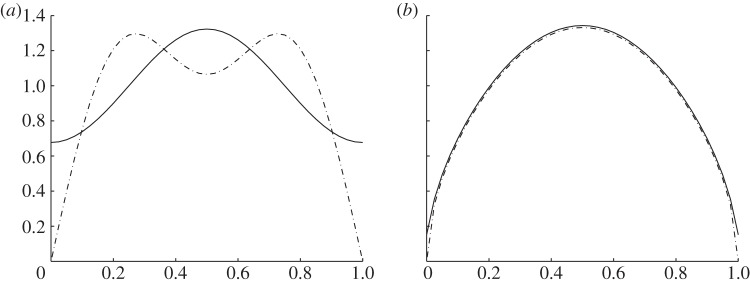
Dependence on number of terms in Fourier series for *u*_0_ at *y*=*a*. Solid line from *y*≤*a* Fourier series expansion. Dashed line from *y*≥*a* Fourier series expansion. (*a*) *N*=2, (*b*) *N*=40.

This physical interpretation then motivates a Fourier series approach and using a rescaling of lengths and frequencies, 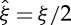, 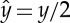, 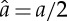, 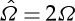 and 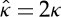 gives the geometry investigated by Evans & Linton [6]. The full Rayleigh–Bloch solution for *u* is obtained as
3.3
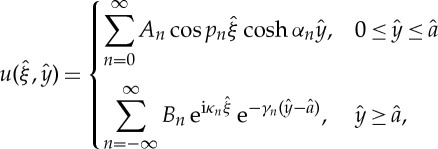
where *p*_*n*_=*nπ*, 
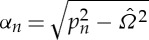
, 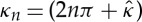 and 
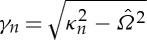
. The coefficients are determined by imposing continuity of *u* and 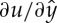 at 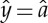, multiplying by 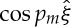 and integrating across the cell width, which allows the *A*_*n*_ to be eliminated and leaves a set of linear equations for the *κ*_*n*_*B*_*n*_ coefficients which are written in matrix notation as
3.4
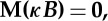
where **M** is a matrix that can be deduced from Evans & Linton [6]. The dispersion relation is obtained by fixing values of 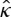 and finding the corresponding values of 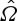 for which 
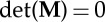
, and then obtaining the eigensolutions for the coefficients *κ*_*n*_*B*_*n*_. The standing wave eigensolution *u*_0_ is the case where *κ*=*π*/2, 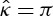, *Ω*=*Ω*_0_, and some of the rows of **M** exhibit singularities. This then requires modifications and the limiting value of the corresponding equations must then be used in place of those of Evans & Linton [6]. Numerically, the infinite summations are truncated for some value of *N* of modes and the infinite summations are replaced by 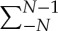 and 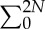. For the standing waves, the *A*_*n*_ are non-zero only for even values of *n*, and the *B*_*n*_ satisfy *B*_*n*_+*B*_−(*n*+1)_=0. As a consequence, *u*_0_ is non-zero on the teeth of the comb for 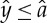, and when repeated in the next strip with a sign change exhibits a discontinuity at 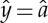. The Fourier series converges to the mid-value, 0, there, but the discontinuity results in Gibb's phenomenon and requires a (fairly) large number of terms, *N*, to be included in the summation to establish continuity of *u*_0_ and 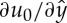 for 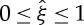 at 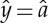. The discontinuity in *u*_0_ at *y*=*a* if insufficient terms are included in the summation is illustrated in figure 4*a*, which shows the profile of *u*_0_ along the *ξ* axis at altitude *y*=*a* for *N*=2, computed with *y*≤*a* (solid line) and *y*≥*a* (dashed line) Fourier series expansions. When *N*=40, shown in figure 4*b*, there is good agreement.

The expansion
3.5
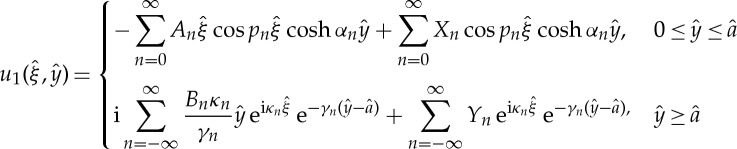
satisfies the differential equation for *u*_1_ (2.6) and the Bloch boundary conditions, for *U*_1_ we again need to choose *κ*=*π*/2. The coefficients *X*_*n*_ and *Y*_*n*_ are to be determined by requiring continuity of *u*_1_ and 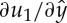 at 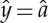. This leads to a matrix equation for the *Y*_*n*_ coefficients
3.6
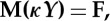
where **M** is the same (singular) matrix as in (3.4) and **F** depends on the known coefficients *A*_*n*_ and *B*_*n*_. Hence, the solution for *u*_1_ is arbitrary with respect to additional multiples of *u*_0_, but these extra terms do not contribute to the coefficient *T* and may be safely ignored. The integrals required to calculate *T* are expressed in terms of the coefficients as:
3.7
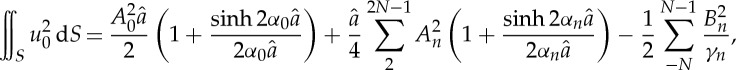
and
3.8
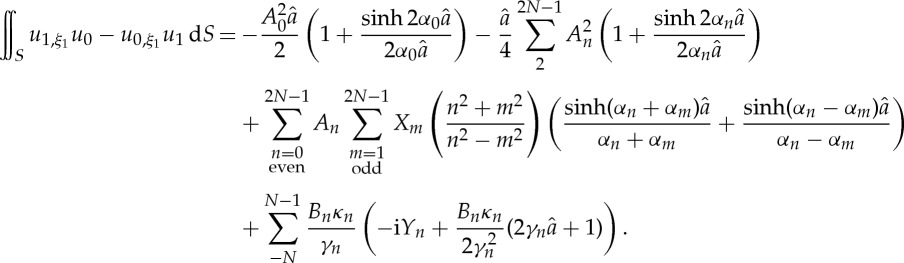


The calculations of the standing wave frequencies and the coefficient *T* are shown in tables 2 and 3 for different values of *N* between 2 and 40 and demonstrate that these values are relatively insensitive to the value of *N* used. There is good agreement with the values obtained from the full numerical simulation and the asymptotic approximation.

**Figure 5.**
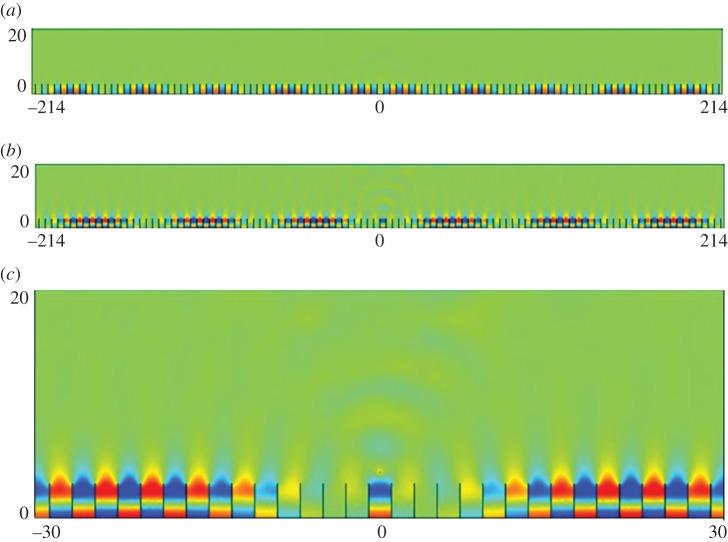
Plots of ℜ(*u*) from finite-element simulations for a comb grating with *a*=3 (cf. figure 2*b*): (*a*) fields generated by a line source with *Ω*=0.4509 (*Ω*_0_=0.45127); (*b*) fields generated by a line source with *Ω*=1.3138 (*Ω*_0_=1.31510) and (*c*) detail of ℜ(*u*) at *Ω*=1.3138. (Online version in colour)

**Figure 6.**
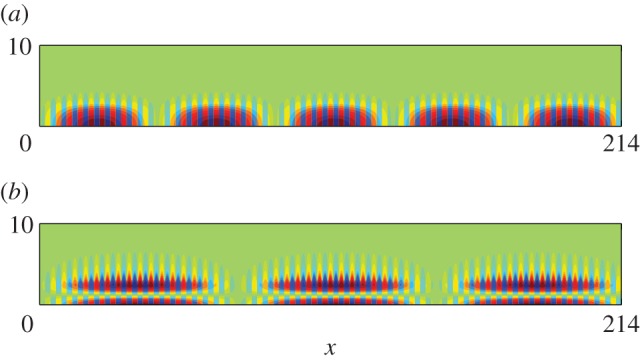
Plots of ℜ(*u*) from HFH for a comb with *a*=3 (cf. figure 5), generated by a line source at (*a*) *Ω*=0.4509 and (*b*) *Ω*=1.3138, respectively. (Online version in colour)

In table 1, we give the values of *T* used in equation (2.19), which combined with the standing wave (*Ω*_0_) and excitation (*Ω*) frequencies yield an effective medium equation. Figure 5 shows the appearance of a new length scale when a periodic comb-like structure with *a*=7, is excited with a line source at the frequencies of *Ω*=0.4509 and *Ω*=1.3138, respectively, in figure 5*a*,*b*. The standing wave eigensolutions closest to these frequencies are shown in figure 3*a*,*b* and show that on the microscale one expects no oscillation or one oscillation along the open waveguide formed by the comb teeth in one strip and this local behaviour is indeed seen in figure 5. There is also clearly a long-scale oscillation along the comb and the calculation of the apparent pseudo-wavelength is possible by HFH as explained in §2*b* and yields the respective wavelengths *λ*/2∼43.4 and *λ*/2∼73. These are in accordance with panels (*a*) and (*b*) of figures 5 and 6 where the latter shows a complete reproduction by HFH of the numerical results, obtained by plotting ℜ(*u*).

### (b) Array of cylinders

Similar to the comb structure, one can also have a diffraction grating constructed from a linear array of obstacles where surface wave modes can again occur. We consider a linear periodic array of cylinders, as in say Evans & Porter [46], where Rayleigh–Bloch modes are observed.

**Figure 7.**
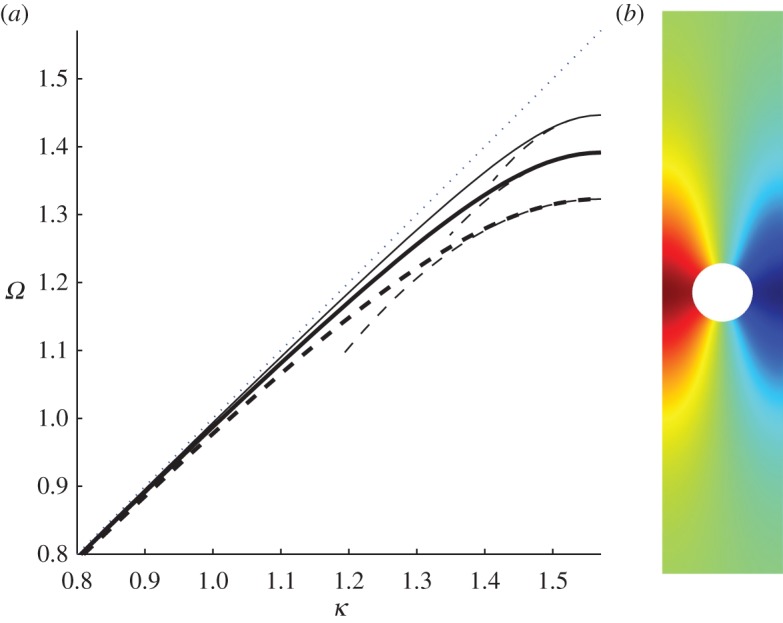
The dispersion branches, for the symmetric mode, (*Ω*=*κ* as dotted line) are shown in (*a*) for cylinders with radii of 0.7 (dashed bold), 0.5 (bold) and 0.4 (solid). The corresponding asymptotic curves, from (2.16), are shown as dashed lines. (*b*) Shows the standing wave eigensolution *U*_0_ for *r*=0.5. (Online version in colour)

**Figure 8.**
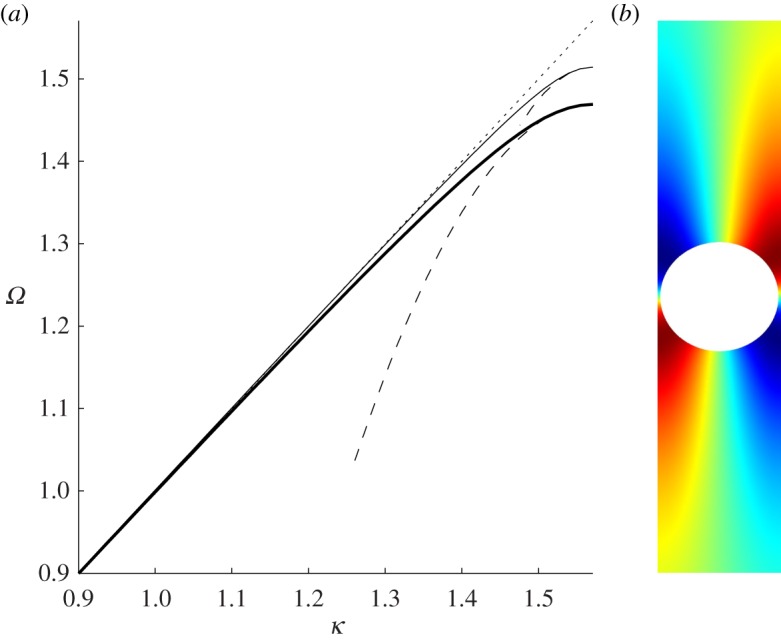
The dispersion branches, for the antisymmetric mode, (*Ω*=*κ* as dotted line) are shown in (*a*) for cylinders with radii of 0.95 (solid line) and 0.99 (bold solid line). The asymptotics are shown as dashed lines. In (*b*), the antisymmetric eigensolution *U*_0_ is shown for *r*=0.95. (Online version in colour)

**Figure 9.**
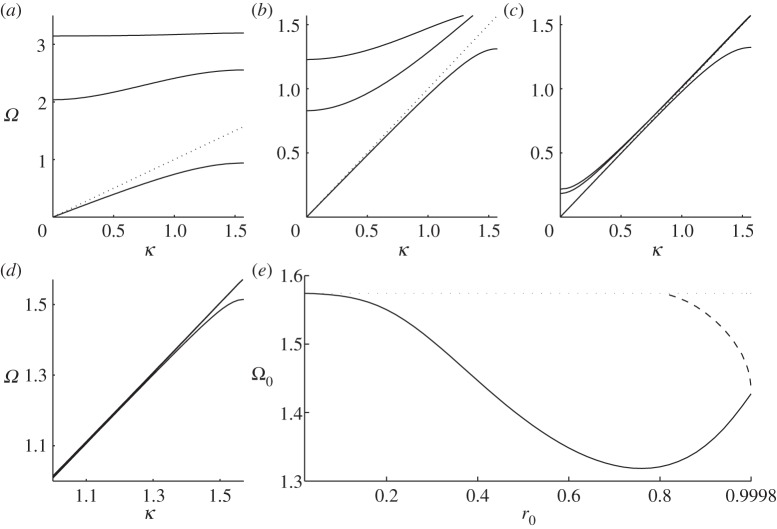
The dispersion curves for cylinders placed in a rectangular array are shown for Bloch waves in the *x*_1_-direction. The lowest three dispersion branches, as solid lines, and *Ω*=*κ* as dotted, are shown in (*a*), (*b*) and (*c*) for rectangle heights of 2, 6 and 30, respectively, where the cylinder radius is 0.95. (*d*) Shows the dispersion curves for a cell of height 30, in solid the second modes for the respective radii of 0.4 and 0.95 and the light-line in dashed. (*e*) Shows the variation of standing wave frequencies for the symmetric (solid) and antisymmetric (dashed) modes versus cylinder radius for the infinite strip.

The first mode, which is symmetric about *y*=0, is shown in figure 7*b* and exists for all radii *r*_0_ of the cylinders such that *r*_0_∈]0,1[. Figure 7*a* shows the dispersion branches for radii *r*_0_=0.4, 0.5 and 0.7 and the associated HFH asymptotics. If the radius is greater than *r*_0_∼0.81, then a second Rayleigh–Bloch mode appears, illustrated in figure 8*b*, which is antisymmetric about *y*=0. To motivate how this occurs, we turn to a two-dimensional rectangular lattice of cylinders, as a generalization of Antonakakis *et al.* [26], so instead of a grating we consider the dispersion diagram of a doubly periodic structure where the width of the rectangles is fixed to 2, and the height is gradually increased until a grating-like strip is obtained. Figure 9*a*–*c* shows the first three modes, and the light-line *Ω*=*κ*, for the respective cell heights of *h*=2, 6 and 30 and each with a centred hole of radius *r*_0_=0.95. Both dispersion modes initially above the light-line converge to the latter as the height of the cell increases and eventually one emerges beneath it. Upon inspection the Bloch mode, for the rectangular array, that passes beneath the light-line has the appropriate symmetry and limits to the antisymmetric mode for the grating. As discussed in Evans & Porter [46], the critical radius value is ∼0.81 and beyond this there is the emergence of the antisymmetric trapped mode; this is illustrated in figure 9*d* which shows the antisymmetric mode, for a rectangular array height of *h*=30, for radii *r*_0_=0.4, 0.81 and 0.95, respectively. For radii *r*_0_=0.4 and 0.81, the mode merges with the light-line, but the mode related to 0.95 emerges below the light-line and one then observes this antisymmetric Rayleigh–Bloch mode. For all radii less than ∼0.81 all modes, bar the first, will collapse on the light-line. Figure 9*e* provides a summary of the variation of the standing wave frequencies with radius, and the appearance of this antisymmetric mode for radii in the interval [0.81,1] is evident. At *r*_0_=1 is a degenerate case, and we stop our calculation at *r*_0_=0.9998. Notably, the asymptotic HFH theory captures the behaviour of the dispersion curves for the antisymmetric case too as shown in figure 8*a*.

**Figure 10.**
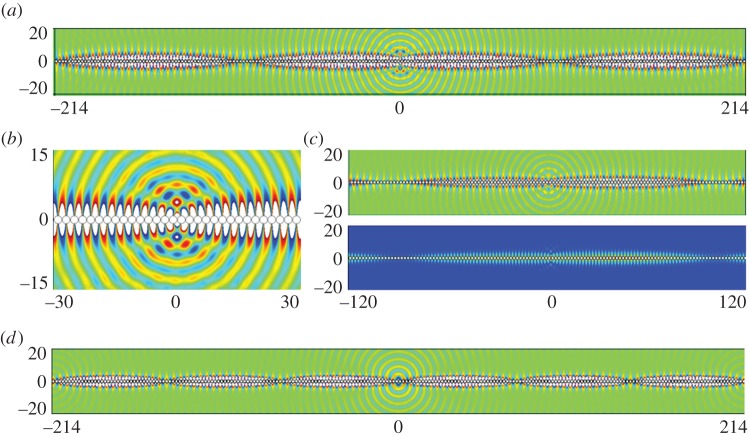
Plots of ℜ(*u*) for a diffraction grating consisting of cylinders of radius *r*=0.95: (*a*) antisymmetric fields generated by a dipole source for *Ω*=1.508 (*Ω*_0_= 1.51445); (*b*) detail close to the dipole source showing the microscale asymmetry; (*c*) detail of the real part (upper panel) and absolute value (lower panel) of the antisymmetric *u* at *Ω*=1.508; (*d*) symmetric fields generated by a line source at frequency *Ω*=1.38 (*Ω*_0_=1.38407). (Online version in colour)

**Figure 11.**
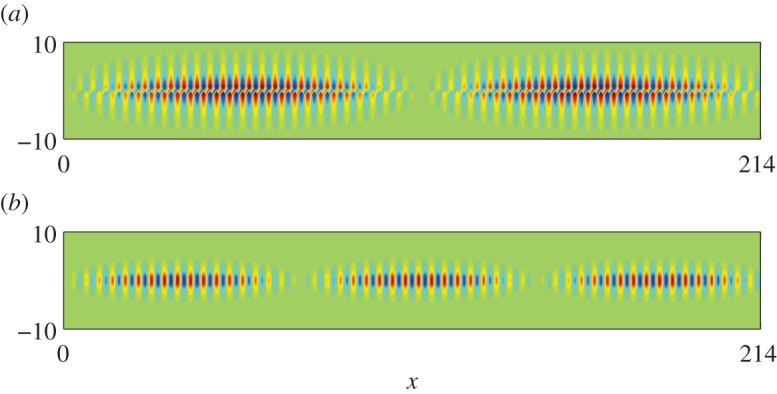
Plots of ℜ(*u*) from HFH for a cylinder of radius *r*=0.95 (cf. figure 10), generated by a line source at (*a*) *Ω*=1.508 and (*b*) *Ω*=1.38, respectively. (Online version in colour)

To illustrate further HFH and the emergence of the long-scale oscillation, we performed large-scale finite-element simulations summarized in figure 10. The antisymmetric mode was generated using a dipole source, to trigger the asymmetry, and the symmetric mode using a line source. The selected frequencies are slightly away from the standing wave frequencies and are, respectively, *Ω*=1.508 and *Ω*=1.38 for panels (*a*) and (*b*). Once again, the apparent length scales are evaluated by HFH to be *λ*/2=109.4 and *λ*/2=72.7 which are confirmed by the numerics as well as in figure 11.

### (c) Array of split ring resonators

**Figure 12.**
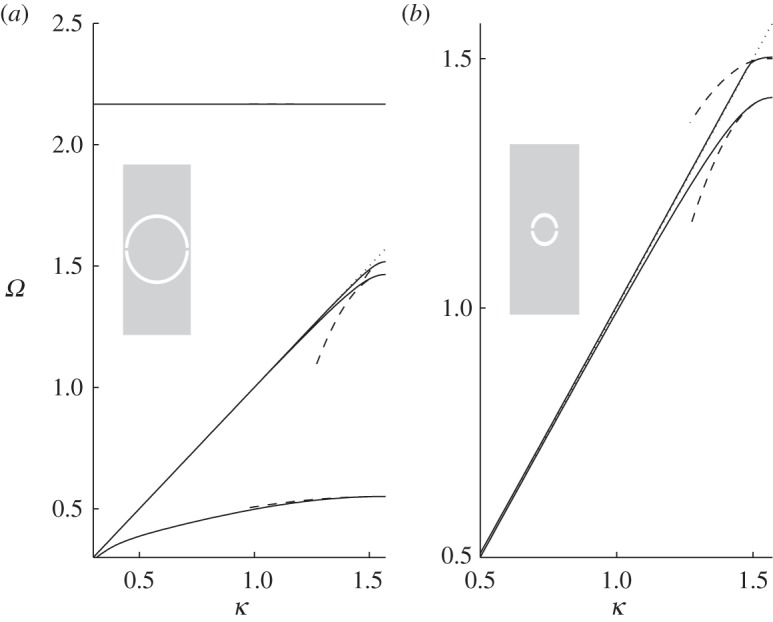
The dispersion branches for the SRR structure. Results from numerical simulations shown as solid lines, the asymptotics as dashed line and the light-line is dotted. (*a*) Is for a large SRR of outer radius *R*_out_=0.95 and inner radius *R*_in_=0.85 and (*b*) is for a smaller SRR with *R*_out_=0.4 and *R*_in_=0.3.

**Figure 13.**
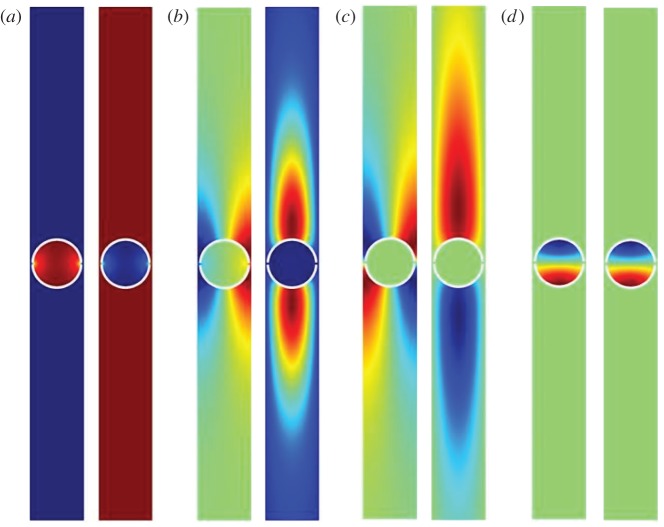
The eigenfunctions *U*_0_, and *U*_1_ shown for the SRR structure, with outer, inner radius *R*_out_=0.95, *R*_in_=0.85 (cf. figure 12*a*). These are for the *Ω*_0_ in table 4 with (*a*–*d*) for ascending *Ω*_0_. In each panel *U*_0_ is shown on the left and *U*_1_ on the right. (Online version in colour)

A left-handed material is an artificial structure that has a negative refractive index over a certain range of frequencies. SRRs, which were introduced by Pendry *et al.* [47] are extensively used to achieve artificial magnetism in metamaterials [48]. Slow backward and fast forward waves have been experimentally observed in chains of SRRs [49], which further motivates the analysis of SRR gratings. For SRRs here, we choose to use a simple cylindrical annulus with two ligaments connecting the inner cylinder to the outer material. The weak coupling between the inner cylinder through these two thin ligaments is important as this arrangement can act as local resonators and this microresonance is important in photonic applications and in metamaterials [47]. In SRR gratings, Rayleigh–Bloch modes occur at frequencies above the cut-off owing to this resonance behaviour within the inner part of the SRR as shown in the fourth mode of figure 12*a* and the resonance is clear in the eigensolution shown in figure 13*d*. The ultra-flat dispersion curve, figure 12*a*, is associated with dipole localized modes in every SRR of the grating and it can be predicted using a geometrical asymptotic technique discussed in Antonakakis *et al.* [26] and Movchan & Guenneau [50].

The modes that arise for the grating of SRR split into two families, one which is very similar to those of the cylinders of the last section, that is, figure 13*b*,*c* are, respectively, similar to those of figures 7*b* and 8*b*. The lowest mode, whose eigensolution is shown in figure 13*a*, is again one primarily associated with the inner cylinder and vibrations of the ligaments.

HFH is used to generate the asymptotics and table 4 shows the standing wave frequencies and respective values of *T* for the first four modes of an SRR grating with outer radius of *R*_out_=0.95. The asymptotics of the dispersion curves again show pleasing accuracy.

**Table 4.** The four standing wave frequencies, below the cut-off, for an SRR grating with *R*_out_=0.95 and *R*_in_=0.85 (cf. figure 12*a*), together with associated values for *T*.

*T*	*Ω*_0_
−0.145181377783699	0.550884858382472
−12.037628085319582	1.465518146041600
−25.144195967619925	1.517732423423271
−0.003773469173013	2.167224509645187

**Figure 14.**
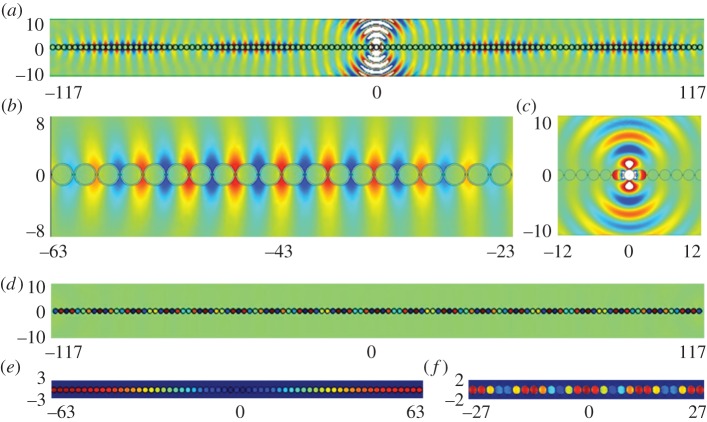
Plots of ℜ(*u*) for an SRR grating with SRR of inner and outer radii 0.85 and 0.95, respectively, and ligaments of thickness 0.06 (standing wave frequencies in table 4): (*a*) field generated by a line source at *Ω*=1.45; (*b*,*c*) detail of ℜ(*u*) at *Ω*=1.45 centred around *x*=−43 showing the developed field (*b*) and around the source (*c*); (*d*) field generated by a line source at *Ω*=0.54; (*e*,*f*) close-up on the absolute value of field *u* at frequency *Ω*=0.55,*Ω*=0.54, respectively. (Online version in colour)

**Figure 15.**
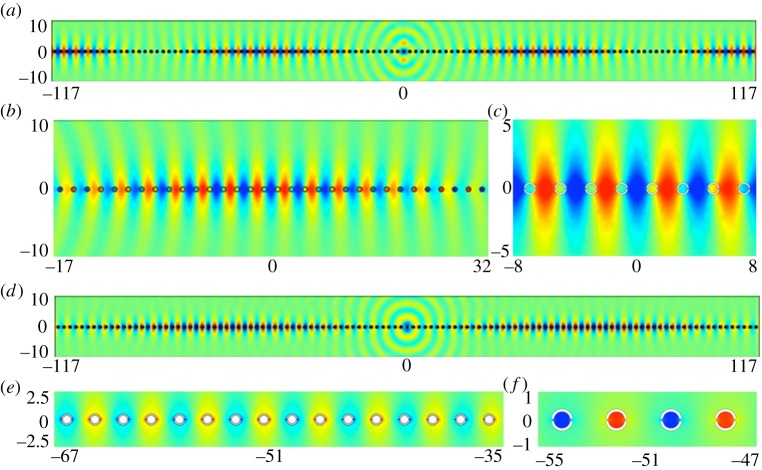
Plots of ℜ(*u*) for an SRR grating with SRR of inner and outer radii 0.3 and 0.4 and ligaments of thickness 0.06: (*a*) fields generated by a line source at *Ω*=1.50 (*Ω*_0_=1.50295); (*b*,*c*) detail of *u* at *Ω*=1.50 centred around *x*=49 showing the developed field; (*d*) field generated by a line source at *Ω*=1.42 (*Ω*_0_=1.42199); (*e*,*f*) detail of ℜ(*u*) at frequency *Ω*=1.42. (Online version in colour)

**Figure 16.**
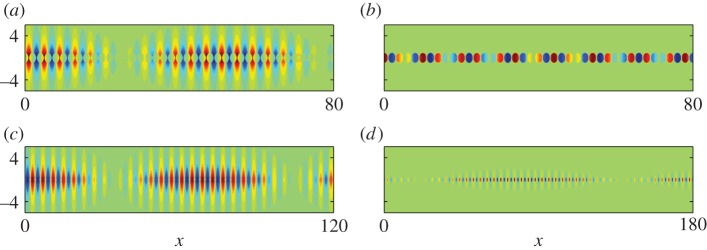
(*a*,*b*) Show ℜ(*u*) from HFH for an SRR, with inner and outer radii of *R*_in_=0.85 and *R*_out_=0.95 (cf. figure 14), generated by a line source at *Ω*=1.45 and *Ω*=0.54, respectively. (*c*,*d*) Show ℜ(*u*) from HFH for an SRR, with inner and outer radii of *R*_in_=0.3 and *R*_out_=0.4 (cf. figure 15), generated by a line source at *Ω*=1.50 and *Ω*=1.42, respectively. (Online version in colour)

Numerical finite-element solutions for line source excitation show plainly this separation into exterior modes akin to those of the cylinder (figure 14*a*–*c*) and those localized almost entirely within the SRR as in figure 14*d*–*f*: in these latter cases, the array acts very clearly as an oscillating string. The smaller SRR illustrated in figure 15 gives an even more pronounced locally anti-periodic oscillation with long-scale oscillation. The excitation frequencies are chosen to be close to those of standing waves and the long-scale behaviour extracted using HFH as seen in the figure 16. The wavelengths associated with figure 16 are in the panel's order of appearance, *λ*/2=51.2,11,69.6,119.1.

## 4. Defect states in quasi-periodic gratings

The previous examples illustrate HFH for perfectly periodic media but its applications go further than this. In §3, HFH asymptotics and the resulting effective media successfully homogenize perfect periodic arrays, but one could also obtain analytical or numerical solutions fairly quickly at least for simple geometries. The real power of HFH lies in its capability to move away from perfect periodicity, we now take the comb of §3*a*, but now vary the height of the comb's teeth with respect to the *x*_1_ coordinate by a function *g*(*X*), so that their height is *a*(1−*ϵ*^2^*g*(*X*)) and address the question of whether localized states exist at specific frequencies in such quasi-periodic media, that is, are there finite energy states that have exponential decay along the array? We make the following change of coordinates in order to transform the varying tooth height in *x*_2_ to constant height pins in the new coordinate *ξ*_2_ such that
4.1
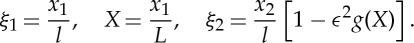
This sleight of hand transforms the medium and moves the tooth heights to a constant within this transformed medium. Following through the asymptotic procedure, as in §2, we obtain three equations ordered in *ϵ*, the only change is at second order, where (2.7) becomes
4.2
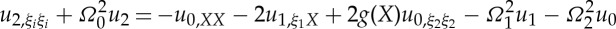
which contains an additional term. Neumann boundary conditions remain unchanged for leading and first order but in second order yield,
4.3
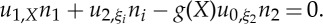
Using a solvability condition, we obtain an equation for *f*_0_ as,
4.4
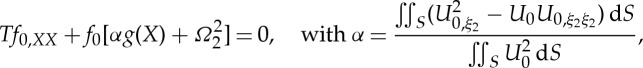
where *T* is given in (2.15). This is a Schrödinger equation and for specific choices of *g*(*X*) exact solutions exist notably for *g*(*X*)=−sech^2^*X* as in Infeld & Hull [51] and Craster *et al.* [29], hence adopting this variation an asymptotic value of the lowest defect mode frequency is explicitly
4.5
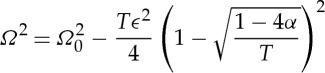
provided that *α*/*T* is always negative, which occurs as *T* is always negative and *α* positive. The associated solutions for *f*_0_(*X*) are [51],
4.6
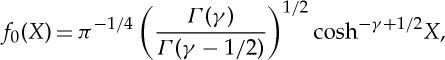
where for the lowest defect mode 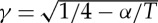 and *Γ*(*γ*) is the Gamma function [52].

**Figure 17.**
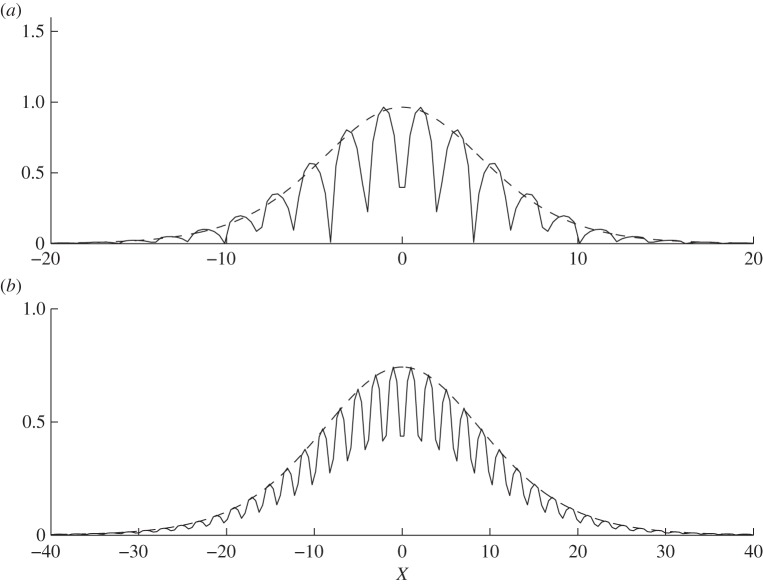
The localized defect mode shown for the comb-like structure with variation of tooth height for *a*=7. The variation follows equation (4.1) with *ϵ*=0.125 in (*a*) and *ϵ*=0.0625 in (*b*). In solid are solutions from FEM simulations of *u* along *x* and for *y*=7, and in dashed are solutions from equation (4.6).

For *a*=7, table 5 shows the predictions of the frequencies at which these defect states arise versus values extracted from finite-element simulations which are reassuringly accurate, and these defect mode frequencies are above the standing wave frequencies as one would expect. The eigenvalues obtained by finite-element simulations are real and show virtually no traces of small imaginary parts and this is in line with the expectation that these are isolated embedded eigenvalues created by perturbing the periodic structure. Perhaps more compelling are the illustrative solutions shown in figure 17, which show *f*_0_ versus the numerical eigensolutions; as both solutions are arbitrary to within a multiplicative constant we normalize to have 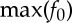 equal to the maximum value from the numerics.

**Table 5.** The predicted frequencies of the localized defect mode near the first standing wave frequency (*Ω*_0_=0.210161050669707; cf. table 1) for the comb-like structure with *a*=7 (cf. figure 2*d*). The frequencies *Ω*_HFH_ come from the asymptotics (4.5), whereas *Ω*_num_ gives predictions from FEM simulations. The parameter *ϵ* controls the variation of tooth height in (4.1).

*ϵ*	*Ω*_HFH_	*Ω*_num_
0.125	0.21247	0.21252
0.0625	0.21074	0.21079

## 5. Concluding remarks

It is shown here that one can take a microstructured surface, or diffraction grating [40,53], and close to the standing wave frequencies that occur, one can represent the surface as an effective string or membrane. The standing waves can occur at high frequencies and as a result the effective stiffness (or permittivity in optics) is not simply an average but involves the integrals over a microscale, importantly the effective equation is posed entirely on the long-scale with the short-scale built in through integrated quantities. Thus, we extend homogenization in two distinct directions enabling microstructured surfaces, instead of the more usual bulk media, to be modelled and away from the usual low-frequency limit. Given the effective equation description, one can then concentrate numerical efforts on modelling instead of capturing the fine scale detail. Indeed, as shown in §4, one can use the effective description to capture analytically features such as defect states caused by non-periodic behaviour.

There are several practical directions that could be pursued using this analysis, notably the surface wave for line source excitation demonstrates the two-scale behaviour beautifully with a short-scale oscillation from one neighbouring strip to the next and, in some sense, chooses its own longer wavelength. The current theory neatly encapsulates this, and this information could be used as part of an inverse problem to determine the quality of microscale or nanoscale surfaces, and the defect states could identify local damage. Importantly, questions related to tuning a surface to have designer properties can be encapsulated into how the coefficient *T* behaves and that too avoids lengthy computations using numerical methods for gratings such as Fourier [54] or differential [55] methods.

In summary, one can now take a microstructured surface, or diffraction grating, that is periodic, or nearly so, and replace it by a continuum description that captures the surface Rayleigh–Bloch waves in the present case, anti-plane shear in acoustics or transverse electrics in optics. Similar phenomena can be investigated with HFH in hydrodynamics and elastodynamics.
